# Digitale Gesundheitsanwendungen zur Behandlung der posttraumatischen Belastungsstörung: Status quo, Potenziale und Herausforderungen

**DOI:** 10.1007/s00115-025-01866-y

**Published:** 2025-07-31

**Authors:** Lena Sophia Steubl, Jan Philipp Klein, Harald Baumeister

**Affiliations:** 1 Abteilung Klinische Psychologie und Psychotherapie, Institut für Psychologie und Pädagogik, Universität Ulm, Ulm, Deutschland; 2 Standort Mannheim-Heidelberg-Ulm, Deutsches Zentrum für Psychische Gesundheit (DZPG), Ulm, Deutschland; 3 Klinik für Psychiatrie und Psychotherapie, Universität zu Lübeck, Lübeck, Deutschland

**Keywords:** Versorgung, Psychotherapie, Internet- und mobilbasierte Interventionen, Früherkennung, Evidenz, Care, Psychotherapy, Internet- and mobile-based interventions, Early detection, Evidence

## Abstract

**Hintergrund:**

Trotz der hohen Prävalenz der posttraumatischen Belastungsstörung (PTBS) steht bisher keine digitale Gesundheitsanwendung (DiGA) zur Behandlung der Erkrankung zur Verfügung.

**Fragestellung:**

Überblick über Möglichkeiten digitaler Interventionen für die Behandlung der PTBS und mögliche Herausforderungen in Bezug auf die Entwicklung einer wirksamen DiGA.

**Material und Methoden:**

Narrative Übersichtsarbeit aktueller Literatur.

**Ergebnisse:**

Internet- und mobilbasierte Interventionen für PTBS haben das Potenzial, eine wirksame Behandlungsoption darzustellen, was eine Reihe von Metaanalysen im Vergleich zu Wartelistenkontrollgruppen, zu Bedingungen mit keiner oder minimaler regulären Versorgung sowie zu aktiven Kontrollgruppen (z. B. Psychoedukation) zeigt. Für die Zielgruppe bestehen jedoch spezifische Herausforderungen, die über allgemein bekannte Barrieren hinausgehen – etwa besondere Bedenken von Hersteller:innen und Behandler:innen hinsichtlich der Sicherheit und eine potenziell noch geringere Adhärenz von Patient:innen.

**Schlussfolgerungen:**

Die Entwicklung von DiGAs könnte perspektivisch auch für die Behandlung der PTBS von Nutzen sein, jedoch müssen die genannten Herausforderungen bei der Gestaltung und Implementierung berücksichtigt werden, um eine wirksame Anwendung zu gewährleisten.

**Zusatzmaterial online:**

Die Online-Version dieses Beitrags (10.1007/s00115-025-01866-y) enthält eine Übersicht über die Begriffsdefinitionen.

## Abstract (en)

**Background:**

Despite the high prevalence of posttraumatic stress disorder (PTSD), no digital health application (*digitale Gesundheitsanwendung*, DiGA) prescribable within the German statutory health insurance system is currently available to treat the disorder.

**Objective:**

Overview of digital intervention options for the treatment of PTSD and potential challenges in developing an effective DiGA.

**Material and method:**

Narrative review of the current literature.

**Results:**

Internet- and mobile-based interventions for PTSD have the potential to serve as an effective treatment option, as demonstrated by a series of meta-analyses comparing them to waiting list control groups, conditions involving no or minimal usual care, and active control groups (e.g., psychoeducation); however, specific challenges exist within the target population that go beyond commonly known barriers, such as pronounced concerns of developers and medical professionals regarding safety and potentially even lower adherence of patients.

**Conclusion:**

The development of DiGAs for the treatment of PTSD might be perspectively promising but the aforementioned challenges must be considered in the design and implementation to ensure an effective application.

Zum gegenwärtigen Zeitpunkt (Stand: März 2025) befinden sich 59 vorläufig und dauerhaft aufgenommene digitale Gesundheitsanwendungen (DiGAs) im Verzeichnis des Bundesinstituts für Arzneimittel und Medizinprodukte [9]. Es steht jedoch keine DiGA zur Behandlung der posttraumatischen Belastungsstörung (PTBS) zur Verfügung, obwohl es sich um eine häufige und schwerwiegende psychische Erkrankung handelt [8, 41].

## Hintergrund zur PTBS

Die posttraumatische Belastungsstörung (F43.1) gehört in der International Statistical Classification of Diseases and Related Health Problems 10 (ICD-10) gemeinsam mit der akuten Belastungsreaktion (F43.0) und den Anpassungsstörungen (F43.2) zu den „Reaktionen auf schwere Belastungen und Anpassungsstörungen“ (F43; [42]). Sie wird durch die Entstehung nach einem Ereignis von außergewöhnlicher Bedrohung oder katastrophalem Ausmaß sowie durch anhaltendes Wiedererleben des Ereignisses, Vermeidung von Gedanken, Gefühlen oder Erinnerungen und eine erhöhte Erregung (Hyperarousal) gekennzeichnet [42]. Im Diagnostic and Statistical Manual of Mental Disorders 5 (DSM-5) wird die PTBS ähnlich kategorisiert [1]. In der ICD-11, die offiziell seit 2022 international in Kraft ist, gibt es eine eigene Diagnosegruppe der belastungsbezogenen Störungen, zu denen neben der PTBS (6B40) auch die komplexe PTBS (kPTBS; 6B41), die anhaltende Trauerstörung (6B42) sowie die Anpassungsstörung (6B43) gehören [43].

Die Prävalenzraten für das Erleben eines potenziell traumatisierenden Ereignisses sowie die tatsächliche Entwicklung einer PTBS unterscheiden sich je nach Population und zugrunde liegendem Klassifikationssystem. Studien aus Deutschland gehen von Raten zwischen 18 und 28 % für das Erleben eines potenziell traumatisierenden Ereignisses aus [25, 29]. Sekundärdaten einer deutschen Krankenkasse ergaben 2017 eine PTBS-Diagnose-Prävalenz von 0,7 % basierend auf der ICD-10 [11]. Basierend auf der ICD-11 werden 1‑Monats-Prävalenzen von 1,5 % für die PTBS und 0,5 % für die kPTBS berichtet [26]. Unter Geflüchteten ist von höheren Raten auszugehen [18].

## Behandlung der PTBS

Die aktuelle deutsche Behandlungsleitlinie für PTBS empfiehlt vorrangig eine traumafokussierte Psychotherapie [33]. Dazu gehören die traumafokussierte kognitive Verhaltenstherapie (TF-KVT) sowie „eye movement desensitization and reprocessing“ (EMDR; [33]). Erstere basiert dabei auf der KVT und beinhaltet z. B. imaginative und narrative Exposition sowie Exposition in vivo und kognitive Umstrukturierung [33]. Bei der EMDR hingegen wird bilaterale Stimulation, meist durch Augenbewegungen, eingesetzt, um belastende Erinnerungen neu zu verarbeiten und ihre emotionale Intensität zu reduzieren [33]. Insgesamt zeigen psychotherapeutische Behandlungen für die PTBS hohe Effektstärken (z. B. standardisierte mittlere Differenz [SMD] = −1,08 für EMDR und SMD = −1,27 für expositionsbasierte KVT; [5, 13, 39]). Psychopharmakologische Ansätze sollen den Leitlinien nach aktuell weder als alleinige noch als primäre Behandlung genutzt werden [33]. Falls trotzdem eine Medikation gewünscht wird, werden Sertralin, Paroxetin oder Venlafaxin als mögliche Wirkstoffe genannt [33].

Auch wenn damit ausführliche Behandlungsleitlinien vorliegen, bleibt ihre erfolgreiche Umsetzung in der Versorgung bislang lückenhaft: Nur ein Teil der Betroffenen erhält überhaupt eine Weiterleitung oder Behandlung, empirisch fundierte Verfahren kommen häufig nicht systematisch zum Einsatz und Teile der Betroffenen profitieren nicht von den Ansätzen [13, 14, 16, 39]. Auch in Deutschland können Gründe hierfür z. B. in der Angst vor Stigmatisierung und einem Mangel an adäquaten Angeboten liegen, nicht nur, aber sicherlich auch für Geflüchtete [6, 8, 14, 16, 44]. Die Nutzung digitaler Interventionen könnte helfen, diese Hindernisse zu überwinden [2, 27, 38, 44] und sogar in den Behandlungsleitlinien für die PTBS wird zumindest in Bezug auf das Erreichen von Betroffenen auf das Internet verwiesen [33].

## Digitale Interventionen

Es haben sich in den letzten Jahren viele Wegen eröffnet, um mithilfe digitaler Interventionen die Behandlung verschiedener Erkrankungen zu unterstützen. Hinsichtlich der Behandlung psychischer Erkrankungen spielen vor allem zwei Möglichkeiten eine Rolle:

- die videobasierte Behandlung und
- sog. internet- und mobilbasierte Interventionen (IMIs; [36]).

Während videobasierte Behandlungen einen wirksamen und akzeptierten Zugang zur Behandlung ermöglichen – insbesondere für Personen mit eingeschränkter Mobilität oder in ländlichen Regionen – tragen sie nicht zur Erweiterung der verfügbaren Behandlungskapazitäten bei. Dieses Potenzial zur Skalierung, also zur Versorgung vieler Personen bei vergleichsweise geringem zusätzlichem Ressourcenaufwand, und flexiblen Bereitstellung lässt sich in erster Linie IMIs zuschreiben [36]. Diese können Betroffenen als frei verfügbare Angebote über die App-Stores zur Verfügung stehen oder in Deutschland als sog. digitale Gesundheitsanwendungen (DiGAs) verordnet werden [36]. Erstere sind aufgrund der fehlenden Regulierung vor allem in Bezug auf ihre Sicherheit, ihre oftmals ungeprüfte Wirksamkeit und die zugrunde liegende Evidenzbasis kritisch zu beurteilen [36]. Zweitere stellen hingegen seit dem Inkrafttreten des Digitale-Versorgung-Gesetzes (DVG) im Dezember 2019 ein reguliertes Versorgungsangebot dar, innerhalb dessen Versicherte in den gesetzlichen Krankenversicherungen in Deutschland Anspruch auf eine Versorgung mit DiGAs haben [21]. DiGAs sind dabei in den meisten Fällen therapeutisch unbegleitete IMIs, die neben einer Zertifizierung als Medizinprodukt der Risikoklasse I oder IIa zusätzlich vom Bundesministerium für Arzneimittel und Medizinprodukte (BfArM) geprüft wurden [23]. Inhalte reichen von Psychoedukation bis zu Tagebüchern und Expositionstraining [9].

Das DiGA-Verzeichnis ging mit den ersten zwei verordnungsfähigen DiGAs am 06.10.2020 online [23] und beinhaltet aktuell 59 vorläufig und dauerhaft aufgenommene Anwendungen (Stand: März 2025) [9]. Der größte Teil davon ist mit 28 DiGAs unter der Kategorie Psyche gelistet. Darunter befinden sich in größtem Umfang und zu fast gleichen Teilen DIGAs für die Behandlung von depressiven Störungen (8 verfügbare DiGAs) und Angststörungen (7 verfügbare DiGAs). Weitere Erkrankungen, für die DiGAs verschrieben werden können, sind Schlafstörungen und substanzbezogene Störungen (jeweils 3 verfügbare DiGAs), Schmerzstörungen und Essstörungen (jeweils 2 verfügbare DiGAs) und Vaginismus/Dyspareunie, leichte kognitive Störung und die emotional-instabile Persönlichkeitsstörung vom Borderline-Typ (jeweils eine verfügbare DiGA). Insgesamt stehen 19 der DiGAs als Apps für die gängigen App-Stores (Google Play Store und Apple App Store) zur Verfügung und 20 als Webanwendung. Elf Anwendungen sind sowohl als Webanwendung als auch als App verfügbar. Unter allen verfügbaren DiGAs ist zum gegenwärtigen Zeitpunkt keine, die für die Behandlung der PTBS oder einer anderen Traumafolgestörung zugelassen ist.

Trotzdem sind DiGAs für die Behandlung der PTBS nicht grundsätzlich ausgeschlossen, denn im wissenschaftlichen Bereich gibt es bereits vielversprechende Forschung zur IMIs für die Behandlung der PTBS.

## Wirksamkeit von IMIs zur Behandlung der PTBS

Eine Reihe von Studien hat bereits die Wirksamkeit von (vorrangig KVT-basierten) IMIs für die Behandlung der PTBS untersucht und Evidenz für deren Wirksamkeit gefunden [35, 37]. So zeigten Metaanalysen moderate Effekte (g = 0,71; elf Studien) hinsichtlich der Symptomreduktion im Vergleich zu passiven Vergleichsgruppen (Warteliste oder [weitgehend] keine spezifische Behandlung) sowie geringe Effekte (SMD = 0,36; 21 Studien) im Vergleich zu aktiven Kontrollgruppen (z. B. nur Psychoedukation oder Schreibaufgaben; [35, 37]). Dabei beinhalteten die untersuchten IMIs hauptsächlich Inhalte zur Psychoedukation und kognitiven Umstrukturierung, aber auch Elemente zur imaginierten Exposition [35].

Detaillierte Evidenz zum Vergleich von IMIs für PTBS mit traditioneller Vor-Ort-Behandlung fehlt allerdings weitgehend. Zusätzlich haben die genannten Metaanalysen, wenn überhaupt, nur wenige Studien zu mobilbasierten Interventionen (i.e. Smartphone-Apps) eingeschlossen, während die im DiGA-Verzeichnis gelisteten Anwendungen wie beschrieben nahezu gleich zwischen mobilbasierten und webbasierten (i.e. browserbasierten) Interventionen aufgeteilt sind [37]. Eine Metaanalyse, die lediglich therapeutisch unbegleitete Apps zur Behandlung der PTBS einschloss, zeigte in Prä-post-Vergleichen moderate Effekte auf die Symptomreduktion (g = 0,55), jedoch keine signifikanten Effekte im Vergleich zu Wartelistenkontrollgruppen (g = 0,09; *p* = 0,574; [17]). Dies entspricht Ergebnissen bei anderen psychischen Erkrankungen, die ebenfalls eine geringere Wirksamkeit rein mobilbasierter Interventionen im Vergleich zu webbasierten Interventionen nahelegen [40]. Ein systematischer Überblick über frei verfügbare Apps für PTBS (im Gegensatz zu den zertifizierten DiGAs) zeigt darüber hinaus, dass zwar inhaltlich qualitativ hochwertige Apps zur Verfügung stehen, diese allerdings mit gravierenden Datenschutz- und Sicherheitsbedenken verbunden sind [31]. Korrespondierend mit der geringen Anzahl an Studien zu mobilbasierten Interventionen fand sich in der Überblicksarbeit darüber hinaus nur für eine der 69 eingeschlossenen Apps – entsprechend 1,4 % – wissenschaftliche Evidenz [31].

Es soll dabei allerdings betont werden, dass digitale Interventionen über eine digitale Nachbildung klassischer Vor-Ort-Behandlung hinaus noch weitere Möglichkeiten bieten – z. B. im Kontext von Ansätzen aus dem Bereich der virtuellen Realität (VR). Diese sind für die Behandlung von Angststörungen auch schon als DiGA verordnungsfähig (Invirto; [9]). Auch in Bezug auf die Behandlung der PTBS gibt es vielversprechende Evidenz für die Wirksamkeit, jedoch ist diese durch kleine Stichproben, die hauptsächlich aus männlichen Militärangehörigen und Veteranen bestehen, eingeschränkt [20].

## Früherkennung und Diagnostik

Zusätzlich zur Behandlung einer PTBS können digitale Interventionen auch einen Beitrag zur Früherkennung und Diagnostik leisten. Hier bieten vor allem „ecological momentary assessment“ (i.e. Erfassung von Echtzeitdaten in natürlichen Lebenskontexten) zur kleinschrittigen Diagnostik und Verlaufsbeurteilung und „mobile sensing“ zur passiven Diagnostik vielversprechende Möglichkeiten [7, 27, 30]. Bei letzterem handelt es sich um die – sich bislang überwiegend im Forschungs- oder Entwicklungsstadium befindliche – Nutzung digitaler Technologien, um kontinuierlich Daten über das Verhalten, die Physiologie und die Umgebung von Individuen zu sammeln und so einen Beitrag zum Screening, zur frühen Diagnose und zum Monitoring von Erkrankungen zu leisten [3, 7]. In der Literatur wurde beispielsweise bereits angedacht, Monitoring-Apps nach massentraumatogenen Ereignissen einzusetzen, die die Integration passiver (z. B. Standortdaten, Nutzung von Sprache und Tastatur, soziale Medien) und aktiver Datenquellen (z. B. Sprachprosodie, Gesichtsanalyse) nutzen, um einen digitalen Phänotyp zu erstellen [10]. Mithilfe maschinellen Lernens und künstlicher Intelligenz könnten daraus Algorithmen für die frühzeitige Erkennung und Intervention entwickelt werden [10].

Während derartige Ansätze vielversprechend erscheinen, ist jedoch noch erhebliche Forschungsarbeit erforderlich, um die notwendigen Modelle aufzustellen, unterschiedliche Ergebnisse zu integrieren, klinische Risiken besser zu verstehen und Bedenken der potenziellen Nutzer:innen zu adressieren (z. B. hinsichtlich Datenschutz; [3, 10]).

## Mögliche Gründe für das Fehlen von DiGAs für PTBS

Dass es trotz dieser Vielzahl an Möglichkeiten bisher keine verfügbaren DiGAs für die Erkrankung gibt, kann verschiedene Gründe haben. Zum einen gilt die PTBS, unter anderem durch die individuellen Traumaerfahrungen, als komplexe Erkrankung und Behandler:innen zeigen auch in der herkömmlichen Behandlung mangelndes Selbstvertrauen in der Durchführung von TF-KVT, negative Einstellungen gegenüber den Methoden und allgemeine Ängste, die sie daran hindern, traumafokussierte Interventionen anzuwenden [12, 28, 32]. Gründe, die dafür aufgeführt werden, sind unter anderem eine Präferenz für individualisierte statt manualisierte Behandlungen und Befürchtungen, Patient:innen zu schädigen [4, 32, 39]. Diese Bedenken und Ängste mögen, neben den auch für andere Erkrankungen geltenden Ansprüchen an wissenschaftliche Evidenz und Datenschutz, in der fehlenden Entwicklung von DiGAs spezifisch für PTBS auch bei potenziellen Hersteller:innen eine Rolle spielen.

Zudem ist für die tatsächliche Nutzung der DiGAs eine Überzeugung der Behandler:innen, die diese verordnen sollen, unerlässlich. Aktuelle Evidenz zeigt, dass insbesondere mangelndes Wissen, begrenzte zeitliche Ressourcen, Unsicherheit im Hinblick auf den klinischen Mehrwert sowie Bedenken hinsichtlich der therapeutischen Beziehung zentrale Barrieren für die Integration neuer Technologien darstellen [15]. Diese Barrieren sind allerdings nicht spezifisch für DiGAs im Kontext von PTBS, sondern reflektieren vielmehr generelle Herausforderungen bei der Implementierung digitaler Interventionen in die psychotherapeutische Regelversorgung. Im spezifischen Kontext von PTBS zeigen sich jedoch zusätzliche Schwierigkeiten, wie Ergebnisse aus bisherigen Studien zur Nutzung von IMIs nahelegen: So wurden in einer Studie von Schulte et al. [34] IMI-Sitzungen zwar als nützlich und verständlich empfunden, aber die in der Behandlung der PTBS besonders relevante Traumaverarbeitung wurde als schwierig durchführbar wahrgenommen.

Außerdem ist bei DiGAs, wie bei allen IMIs, die Nutzungsadhärenz von großer Bedeutung, was für Traumabetroffene aufgrund der Vermeidungs- und Hyperarousalsymptomatik eine hohe Hürde darstellen könnte [44].

Eine Einbindung in eine herkömmliche Behandlung (i.e. „blended therapy“) – etwa im Sinne eines Add-ons zur Unterstützung der Vorbereitung, Durchführung oder Nachbereitung herkömmlicher Interventionen – erscheint damit sinnvoll und notwendig [24]. Allerdings ist diese in der Implementation – gerade auch im fragmentierten DiGA-Kontext, bei dem DiGA-Anbietende und Vor-Ort-Behandler:innen wenig Schnittstellen aufweisen und die Anwendungen in den meisten Fällen therapeutisch unbegleitet sind, – besonders komplex [19].

## Ausblick

Trotz bestehender Herausforderungen deuten erste Studien darauf hin, dass DiGAs perspektivisch auch für die Behandlung der PTBS von Nutzen sein könnten. Für die erfolgreiche Entwicklung und Implementierung in den Versorgungskontext sind dabei mehrere Aspekte zentral.

- Erstens müssen umfangreiche klinische Studien durchgeführt werden, um die Wirksamkeit und Sicherheit der DiGA nachzuweisen – insbesondere bei bislang wenig erforschten mobilbasierten Interventionen, die zunehmend den Nutzungsstandard darstellen.
- Zweitens gilt es, bestehende Herausforderungen im Zusammenhang mit Bedenken seitens der Hersteller:innen und Behandler:innen unter anderem gegenüber manualisierten und leitliniengerechten, insbesondere expositionsbasierten, Behandlungsansätzen zu bewältigen. Hierbei spielt die gezielte Integration von Psychoedukation und Symptommanagement eine entscheidende Rolle, während gleichzeitig eine angemessene therapeutische Begleitung oder Verzahnung mit der Vor-Ort-Behandlung (i.e. „blended therapy“) sichergestellt werden muss.
- Drittens ist die Förderung von Adhärenz und Engagement innerhalb der spezifischen Patient:innengruppe essenziell, um die Effektivität der Interventionen zu gewährleisten. Hier können z. B. regelmäßige Abfragen von Spannungszuständen eine Rolle spielen, aber auch Gamification-Ansätze [22].
- Viertens könnten neue Technologien dazu beitragen, DiGAs für die Behandlung der PTBS weiter zu verbessern. Dabei sollten DiGAs nicht lediglich eine digitale Nachbildung klassischer Vor-Ort-Behandlung darstellen, sondern die spezifischen Potenziale digitaler Ansätze ausschöpfen.

Langfristig könnte so das Konzept der DiGAs einen wertvollen Beitrag zur Versorgung von Patient:innen mit PTBS leisten.

## Fazit für die Praxis

- Es gibt mittlerweile zahlreiche digitale Gesundheitsanwendungen (DiGAs), allerdings ist darunter keine für die Behandlung der posttraumatischen Belastungsstörung (PTBS).
- Studien zur Wirksamkeit internet- und mobilbasierter Interventionen (IMIs) für die Behandlung der PTBS liefern vielversprechende Ergebnisse, allerdings ist die Evidenz spezifisch für mobilbasierte Angebote dünn.
- Besondere Herausforderungen in Bezug auf die Nutzung von DiGAs zur Behandlung der PTBS beziehen sich auf mögliche Bedenken von Hersteller:innen, möglicherweise fehlende Akzeptanz unter Behandler:innen, fragliche Adhärenz von Patient:innen und unklare Einbindung in die Behandlung.

## Supplementary Information

Wichtige Begriffe in der digitalen Gesundheitsversorgung im Bereich Psychiatrie und Psychotherapie
